# Stem Cell‐Based Tissue‐Engineered Laryngeal Replacement

**DOI:** 10.5966/sctm.2016-0130

**Published:** 2016-09-09

**Authors:** Tahera Ansari, Peggy Lange, Aaron Southgate, Karin Greco, Carla Carvalho, Leanne Partington, Anthony Bullock, Sheila MacNeil, Mark W. Lowdell, Paul D. Sibbons, Martin A. Birchall

**Affiliations:** ^1^ Department of Surgical Research, Northwick Park Institute for Medical Research, Harrow, United Kingdom; ^2^ University College London Ear Institute, Royal National Throat Nose and Ear Hospital, London, United Kingdom; ^3^ Department of Haematology, University College London Medical School, London, United Kingdom; ^4^ Department of Material Science and Engineering, University of Sheffield, Sheffield, United Kingdom

**Keywords:** Tissue engineering, Larynx, Animal model, Tissue scaffold, Human stem cell

## Abstract

Patients with laryngeal disorders may have severe morbidity relating to swallowing, vocalization, and respiratory function, for which conventional therapies are suboptimal. A tissue‐engineered approach would aim to restore the vocal folds and maintain respiratory function while limiting the extent of scarring in the regenerated tissue. Under Good Laboratory Practice conditions, we decellularized porcine larynges, using detergents and enzymes under negative pressure to produce an acellular scaffold comprising cartilage, muscle, and mucosa. To assess safety and functionality before clinical trials, a decellularized hemilarynx seeded with human bone marrow‐derived mesenchymal stem cells and a tissue‐engineered oral mucosal sheet was implanted orthotopically into six pigs. The seeded grafts were left in situ for 6 months and assessed using computed tomography imaging, bronchoscopy, and mucosal brushings, together with vocal recording and histological analysis on explantation. The graft caused no adverse respiratory function, nor did it impact swallowing or vocalization. Rudimentary vocal folds covered by contiguous epithelium were easily identifiable. In conclusion, the proposed tissue‐engineered approach represents a viable alternative treatment for laryngeal defects. Stem Cells Translational Medicine *2017;6:677–687*

**Significance Statement**

This report describes the production and in vivo implantation of a biologically derived hemilarynx and subsequent in vivo assessment and analysis under regulated Good Laboratory Practice conditions. This study illustrates how physiological laryngeal‐related functions can be retained.

## Introduction

There are no satisfactory conventional solutions for patients with end‐stage laryngeal structural disorders, which can result from trauma or cancer resection. This has profound impact on quality of life, especially with respect to swallowing, breathing, and speech. Conventional solutions to advanced structural disorders of the larynx are suboptimal. Of the 2,000 individuals diagnosed annually with laryngeal cancer in the U.K., 800 undergo local resection that leaves permanent defects in the vocal cords and hoarseness; the 500 most advanced cases have their larynx removed completely; the remainder undergo chemo‐radiotherapy, which achieves good cure rates but has high morbidity, 5% mortality, and can leave a functionless larynx 1.

A regenerative medicine solution providing anatomical restoration of the larynx would improve the results of resection (e.g., by replacing vocal cord with vocal cord instead of fibrotic scar tissue), avoid some laryngectomies, and lower the threshold for selecting surgery over chemoradiotherapy, thus reducing morbidity. Evidence has shown that most of the larynx can be removed with preservation of vocal and sphincter functions, provided one side retains movement (i.e., the “cricoarytenoid‐nerve‐muscle” unit) 2, 3

Regeneration of the vocal folds by various tissue engineering approaches has been attempted using synthetic hyaluronic acid‐dextran hydrogels 4 (accompanied by a foreign body reaction), hyaluronic acid‐based microgels with a controllable degradation rate 5, and collagen hydrogel composites 6, 7. The consensus is that synthetic or collagen‐based composite scaffolds experience accelerated degradation mediated by fibroblasts, resulting in contraction of the matrix. Biologically derived, decellularized acellular matrices have also been used in a vocal‐fold injury model: Bovine‐derived acellular scaffolds were implanted in a rat model to repair bilateral wounds created in the posterior vocal folds 8. Histological assessment showed an initial infiltration of inflammatory cells and fibroblasts, followed by remodeling of the extracellular matrix (ECM). Although advantageous with respect to mechanical properties, composition, and architecture, these scaffolds are capable of eliciting an immune response that results in a foreign‐body reaction, depending how the initial decellularized scaffold was produced. In humans, small areas of cricoid cartilage have also been replaced using collagen sponge combined with a synthetic mesh 9.

A limited number of tissue‐engineered implants have progressed to late‐phase Good Laboratory Practice studies and fewer to early‐phase clinical trials. The larynx is a good subject for translational stem cell science: There is a defined clinical need, clinical success in a closely related organ (i.e., trachea 10), and the cells used have limited requirements for immediate tissue perfusion. The availability of tissue‐engineered partial laryngeal implants would also transform the treatment of the patients with advanced trauma to the larynx, where long‐term tracheostomy is presently the only option for many. Laryngeal allo‐transplantation may offer an option for some in the future 11, but unlike the treatment proposed here, it requires immunosuppression.

The tissue generated in this study was not fully functional in that we did not replace muscle and nerve. However, the clinical need does not necessarily demand this, because we did not replace the whole larynx. We proposed that this technology would provide functional replacement in terms of airway, voice, and swallowing in a manner superior to that provided by present techniques.

## Materials and Methods

### Regulatory Guidelines

This study was carried out in accordance with the Animal (Scientific Procedures) Act 1986, which conforms to the European Convention for the Protection of Vertebrate Animals Used for Experimental and Other Scientific Purposes. In addition, this study was performed in compliance with the Good Laboratory Practice Regulations 1999 (Statutory Instrument no. 3106) and is in conformity with, and implements the requirements of, European Union directives 2004/09/EC and 2004/10/EC. It was designed after consultation with the U.K. Medicines and Healthcare Products Regulatory Agency to provide data suitable for submission for a Clinical Trials Authorization.

### Study Design

Six female White/Landrace cross‐bred pigs, 3–4 months old, were brought into the facility and allowed to acclimatize for 2 weeks before any surgical procedure. Each animal was given a unique identification number (412–417). The average body weight of the animals at the first surgical procedure was between 43 and 55 kg. Each immune‐suppressed pig received a full‐thickness segment of decellularized larynx in a two‐step procedure directly into a defect created in the larynx. Each decellularized hemilarynx was initially seeded with human mesenchymal stem cells (MSCs) and implanted into a sternomastoid muscle fascial pocket for 1 month (stage 1). Thereafter, the scaffold was relocated into a full‐thickness defect created in the cricoid cartilage. However, at this stage, the luminal aspect of the scaffold was covered with a tissue‐engineered oral mucosal sheet consisting of human oral epithelial cells and oral fibroblasts seeded onto an acellular human dermal matrix (stage 2).

Animals were recovered and monitored for an additional 6 months. One pig was killed early because of an ear infection relating to a long‐term underlying pathology exacerbated by immunosuppression.

### Biological Scaffold

Decellularized pig hemilarynges were produced using a patented protocol developed in‐house. Larynges were harvested from pigs from unrelated studies that were killed to implement refinement, reduction, and replacement (“the 3Rs”).

Each larynx was initially frozen for 24 hours at −20°C and then completely thawed at room temperature. The entire decellularization process was carried out using a small desiccator (Sigma‐Aldrich, Gillingham, Dorset, U.K., http://www.sigmaaldrich.com). To create a vacuum, a desiccator was attached to a Telstar Vacuum Pump 2F‐10 (Pendle Refrigeration Wholesale, Burnley, U.K., http://pendle‐refrig.com) fitted with a digital vacuum gauge (Pendle Refrigeration Wholesale). A vacuum was created to a level of <1 kPa absolute. The desiccator was then placed into a shaking incubator (100 rpm) at either 4°C or 37°C, depending on the temperature required within the protocol. All solutions used during the decellularization process contained 1% penicillin/streptomycin (Sigma‐Aldrich).

Briefly, the thawed tissue was incubated in 50 nmol/L latrunculin B (Sigma‐Aldrich) in high‐glucose Dulbecco's modified Eagle's medium (Thermo Fisher Scientific Life Sciences, Waltham, MA, http://www.thermofisher.com) for 2 hours at 37°C. All further steps were performed at room temperature. Each larynx was washed in distilled water twice for 15 minutes between incubation steps. The tissue was then incubated in 0.6 mol/L potassium chloride (Sigma‐Aldrich) for 2 hours, followed by 1.0 mol/L potassium iodide (Sigma‐Aldrich) for 2 hours, and then left to wash overnight in distilled water. The following morning, the potassium chloride and potassium iodide incubations steps were repeated, followed by incubation in 1 Kunitz unit (KU)/ml DNase I (Sigma‐Aldrich) for 2 hours. After another wash in distilled water, twice for 15 minutes, the tissue was subjected to another freeze (−20°C) and thaw cycle for 24 hours.

Tissue was thawed at room temperature for 24 hours and then incubated in a detergent solution containing 0.25% Triton X‐100 (Sigma‐Aldrich), 0.25% sodium deoxycholate (Sigma‐Aldrich) in phosphate‐buffered saline solution (PBS) at 37°C for 24 hours. The tissue was then washed twice over 48 hours with sterile water (Baxter, Deerfield, IL, http://www.baxter.com) at 4°C. After washing, the tissue was incubated with 2,000 KU/L DNase (Sigma‐Aldrich) and 0.1 g/L RNase (Roche, Basel, Switzerland, http://www.roche.com) at 37°C for 24 hours to solubilize any remaining nuclear content. After a further 24 hours of washing with sterile water (Baxter) at 4°C, the DNase/RNase step was repeated once more. The decellularization protocol was concluded with an intensive wash using sterile water over 48–72 hours. We validated successful decellularization of each larynx by histological and molecular analysis, and biomechanical testing.

### Molecular Analysis

The GenElute mammalian genomic DNA miniprep kit (Sigma‐Aldrich) was used for DNA extraction and quantification following the manufacturer's instructions. The Blyscan GAG assay kit (Biocolor, Carrickfergus, U.K., http://www.biocolor.co.uk) was used to quantify sulfated glycosaminoglycan content of fresh and decellularized larynx samples (muscle and cartilage components separately). The collagen content of fresh and decellularized larynx (cartilage and muscle) was quantified with the Sircol collagen assay kit (Biocolor). A detailed description for each is presented in the supplemental online data.

#### Biomechanics

**Figure 1:**
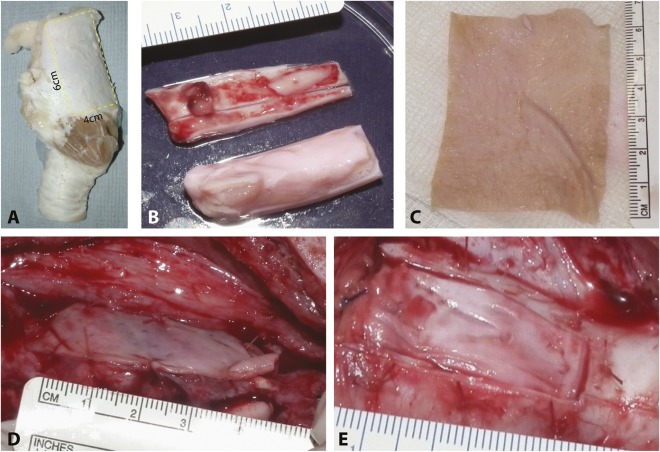
Surgical implantation. **(A):** The size of the implant is illustrated on a nondecellularized hemilarynx. **(B):** Explanted host thyroid cartilage and the implanted piece of decellularized larynx. Tissue‐engineered oral mucosal sheet before implantation **(C)** and sutured onto the luminal aspect of the implanted scaffold at stage 2 **(D)**. **(E):** Closure of the overlying decellularized cricoid cartilage.

The specimens were subjected to uniaxial tension until failure, confirmed by the loss of load and the appearance of tears in the tissue. For each test, a sample of muscle and cartilage from both the fresh and decellularized larynges was used. Specimens, in the form of flat rectangular pieces with a maximum length of 33 mm, were clamped into sample holders and loaded at a constant tension rate of 100 mm per minute and a maximum force of 500 N. The tests were performed with the application of uniaxial tension with an Instron In‐Spec 2200 Benchtop Portable Tester (Instron, Norwood, MA, http://www.instron.us) at room temperature. The tensile tester recorded in real‐time the load and the elongation to which the tissue was subjected. Parameters such as maximum force (N), rupture force (N), and extension at maximum load (mm) were recorded. The ratio of stress to strain was calculated (Young's modulus), which is a measure of the stiffness of an elastic material. After decellularization, the cricoid cartilage component, together with the overlying muscle and mucosal layer, was trimmed to an approximate size of 6 cm × 4 cm × 2 cm (Fig. 1A, 1B).

### Human Bone Marrow‐Derived Mesenchymal Stem Cells

Human bone marrow‐derived mesenchymal stem cells (BM‐MSCs) were obtained from bone marrow aspirates of patients who were donating BM for therapeutic MSC isolation and culture, and who had consented to the use of excess cells for research. They were isolated by plastic adherence before adherent culture in media supplemented with fetal calf serum for a minimum of three and a maximum of seven passages. Cells were split when they were >70% confluent by trypsinization and replating at approximately 1,000 cells per cm^2^. We seeded each hemilarynx by using a custom‐built bioreactor at a seeding density of 1.5 × 10^5^/cm^2^. Human BM‐MSCs were suspended in the smallest volume of medium and seeded directly onto the cartilaginous surface and left to attach for 30 minutes in a humidified CO_2_ incubator. Following attachment, 50 ml of warmed medium was added (sufficient to submerge the seeded scaffold) and left in the humidified CO_2_ incubator until required.

### Tissue‐Engineered Oral Mucosal Sheet

Using a previously published method 12, a sheet of three‐dimensional (3D) tissue‐engineered allogeneic human oral mucosa (approximately 300–600 µm thickness) was produced using split thickness human dermis. Each sheet of donor glycerolized skin (Euroskin) was washed in several changes of PBS before being soaked in 1 M sodium chloride overnight at 37°C followed by mechanical removal of the epidermis. The de‐epidermized dermis (DED) was washed in PBS and then stored at 37°C in medium for 2–3 days to verify sterility. The decellularized human allodermis was then ready to be seeded with human oral keratinocytes (380,000 per cm^2^) and human fibroblasts (125,000 per cm^2^) at 37°C for 2 days in 10% Green's media. The DED and oral cells were cocultured for 14 days at an air‐liquid interface using a floating sterile plastic grid (Fig. 1C) 12.

### Surgical Implantation

#### Stage 1: Intramuscular Implantation of Laryngeal Replacement

One day before the surgical procedure (stage 1), each animal was given the immune‐suppressive drug cyclosporine 10 mg/kg (Neoral; Novartis, Frimley, U.K., https://www.novartis.co.uk), which they continued to receive daily for 80 days. The dose given was based on previous in‐house experience and blood analysis. On the day of surgery, each pig was premedicated intramuscularly with ketamine (5 mg/kg; Zoetis, Florham Park, NJ, https://www.zoetis.com) and xylazine (1 mg/kg; Bayer UK, Newbury, U.K., https://www.bayer.co.uk), both obtained via the National Veterinary Service (Stroke on Trent, U.K.). General anesthesia (GA) was induced with isoflurane over oxygen and nitrous oxide. After intubation, anesthesia was maintained with isoflurane. Cefuroxime 750 mg (Flynn Pharma, Stevenage, U.K., http://www.flynnpharma.com) was given intravenously; subcutaneous analgesia (carprofen 4 mg/kg [Rimadyl]; National Veterinary Services, Stoke‐on‐Trent, U.K., http://www.vetwholesaler.co.uk/), subcutaneous anthelmintic (ivermectin, 1‐ml standard dose; Merial, Athens, GA, http://merial.com), and hypromellose eye drops (FDC International, Dubai, United Arab Emirates, http://www.fdcinternational.com) were given.

The neck was extended and a midline incision (approximately 3–5 cm) was made through the skin from the mandibular region to approximately 15 cm above the manubrium sterni. For pig 412, the right sternomastoid muscle was prepared to provide numerous small feeder vessels from the lateral side. For pigs 413–417, a pocket was created using the sternomastoid muscle and the adjacent fascia. The muscle was prepared to provide an active blood supply and the scaffold was inserted into the pocket such that the seeded side of the scaffold was in contact with the prepared muscle. The pocket was closed using 4‐0 Vicryl sutures (Ethicon UK, Wokingham, U.K., http://gb.ethicon.com). The overlying skin was closed using 2‐0 Prolene sutures (Ethicon UK) and veterinary wound powder (Battles UK) and plastic skin were applied topically.

#### Stage 2: Relocation of Seeded Laryngeal Replacement Into the Larynx

Each animal was given a GA as described. A midline incision using the same approach as used in stage 1 was made. The sternothyroid muscles were separated and an incision made in the thyroid cartilage down to the cricothyroid membrane. A rectangular piece of a cricothyroid cartilage (of a size similar to the implanted scaffold [Fig. 1B]), together with the underlying soft tissue and the tip of the vocal folds, was removed, taking care to leave the remainder of the arytenoid cartilage and the conus elasticus intact. The seeded‐implanted de‐cellularized scaffold was assessed visually, and the luminal side of the scaffold was scraped to create a vascularized interface by making it bleed before a tissue‐engineered oral mucosal sheet was sutured with 4/0 Vicryl (Ethicon UK) to the luminal aspect of the scaffold (Fig. 1D). The entire scaffold was parachuted into place; the mucosal aspect of the graft was sutured to the opposing side with 3‐0 Vicryl sutures (Ethicon UK), taking care not to disrupt the sutured oral mucosal sheet. The cartilage was sutured into place with 4‐0 Vicryl (Ethicon UK) (Fig. 1E) and the skin closed, as described.

### In Vivo Assessment

#### Vocal Recordings

Before any surgical intervention, a 2‐ to 3‐minute vocal recording of each animal was made; this was repeated at 8 weeks and 4 months after stage 2 and immediately before termination of the study. Each recording was made using a handheld Tascam Linear PCM recorder (DR‐07MKII; Tascam Teac Professional, Montebello, CA, http://tascam.com). The data were initially cleaned up using the Audacity software (http://audacityteam.org) such that only clearly identifiable “squeals or grunts” were put forward for further analysis. The final data set from each animal was analyzed for frequency versus intensity of the waveform over the duration of the study for each animal.

#### Bronchoscopic Evaluation

A bronchoscopic assessment under spontaneous ventilation of the luminal aspect of the larynx containing the implanted scaffold was undertaken together with a mucosal brushing for each animal at 2 and 4 weeks and at 2 and 6 months.

#### Blood and Serum Analyses

A blood sample was also taken at 2 and 4 weeks and at 2 and 6 months for each animal for full hematology, biochemistry, and coagulation analyses. Additional blood serum analysis for interleukin (IL)‐6 (porcine specific IL‐6 enzyme‐linked immunosorbent assay [ELISA] kit, catalog no. ab100755; Abcam, Cambridge, U.K., http://www.abcam.com) and IL‐10 (swine‐specific KSC0101; Thermo Fisher Scientific Life Sciences, Waltham, MA, http://www.thermofisher.com) were performed using commercially available ELISA kits, following the manufacturers’ instructions.

#### Mucosal Brushing

To identify the human cells on the tissue‐engineered (TE) oral mucosal sheet, we used two different anti‐human MHC‐1 antibodies (purified HLA‐ABC monoclonal antibody [mAb], clone W6/32, product code 311402, BioLegend, London, U.K., http://www.biolegend.com; and anti‐HLA mAb clone EP1395Y, catalog no. ab 52922, Abcam) and anti‐human α‐GAL (α‐GAL epitope mAb clone M86, catalog no. ALX‐801‐090‐1; Enzo Life Sciences, Exeter, U.K., http://www.enzolifesciences.com). α‐GAL staining was performed under the following parameters: antigen retrieval: proteinase K for 1 minute, nonspecific block with 2% BSA for 30 minutes, and 1:50 dilution for 2 hours. To further characterize the cells, we stained the mucosal brushing with an anti‐human cytokeratin marker (mouse monoclonal anti‐human CK‐7‐clone OV‐TL12/30 [Agilent Technologies, Ely, U.K., http://www.agilent.com] at 1:50 for 30 minutes, no antigen retrieval).

### Explantation of the Scaffold, and Tissue Assessment

#### Explantation and CT Imaging

Following termination, the operative site was carefully dissected and the entire larynx and a portion of the trachea were explanted and CT imaged (Innova 4100; GE Healthcare, Little Chalfont, U.K., http://www3.gehealthcare.com) to produce a 3D rendition of the entire larynx, with the specific aim to locate the implanted decellularized scaffold.

#### Histology

Each larynx was cut longitudinally on the posterior aspect, reflected open to obtain an internal view, and photographed. The entire larynx was placed into 10% neutral buffered formal saline. After adequate fixation, each larynx was bisected (proximal to distal) off the midline on the contralateral side to the implanted scaffold (i.e., on the control side). Each scaffold was cut at 0.5‐cm intervals, starting at the distal (tracheal) end toward the proximal (thyroid cartilage). Each segment was placed into a histology cassette and processed to paraffin wax blocks by routine procedures. Each block underwent microtomy to produce sections 5 µm thick. Sections were stained with hematoxylin and eosin, and Picrosirius red with Miller's elastin.

#### Immunohistochemical Analysis

Tissue sections were stained with following antibodies: α‐smooth muscle actin (1:4,000 for 1 hour; mouse mAb, clone: 1A4, Sigma‐Aldrich); collagen II (catalog no. Abcam 3092 [Abcam]; mouse mAb; antigen retrieval: Dako Target retrieval [Agilent Technologies], incubation 1:100 for 2 hours); collagen X (catalog no. Abcam 49945, mouse mAb [Abcam]; antigen retrieval: pH 6 citrate buffer for 20 minutes, incubation at 1:200 overnight); aggrecan (anti‐aggrecan ARGxx antibody, mAb, clone BC‐3, catalog no. Ab 3773 [Abcam], antigen retrieval: proteinase K for 2 minutes, incubation at 1:200 overnight); and CD 44 (catalog no. Abcam 1119863, rat mAb, antigen retrieval: citrate buffer for 10 minutes, incubation at 1:100 for 1 hour). The protocol can be found in the supplemental online data.

#### In Situ Hybridization for Alu Detection

A commercially available kit was used for the detection of Alu sequence as an indicator for the presence of human cells (Rembrandt In Situ and Hybridization and Detection Universal DISH & HRP detection kit, catalog no. A001K.0101, PanPath, Budel, The Netherlands, http://www.panpath.nl). Details can be found in the supplemental online data.

### Statistical Analysis

Data (biomechanics, molecular data, and blood serum analysis) were calculated as mean ± SE, and significance was determined by performing two‐tailed Student's *t* tests (Prism 5; GraphPad Software, La Jolla, CA, http://www.graphpad.com), followed by Bonferroni as a post hoc test (molecular and blood serum) or analysis of variance (biomechanics). A *p* value of less than .05 was considered significant.

## Results

### Negative Pressure‐Assisted Decellularization of Porcine Larynx

**Figure 2:**
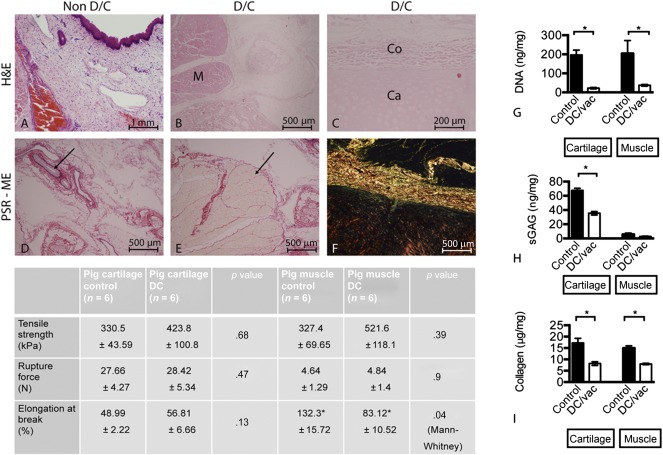
Evaluation of the decellularized porcine larynx using histological, molecular, and biomechanical analyses. Sections were stained with H&E and compared against control tissue **(A)** for the absence of intact nuclear material within muscle **(B)**, cartilage, and overlying collagen **(C)**. Sections were further stained with PSE‐ME to illustrate intact elastin within the blood vessels (arrow) **(D)** and intact collagen fibers between the muscle bundles (arrow) **(E)**. **(F):** Under polarized light, the structural integrity of the collagen was further verified. Molecular analysis for the amount of DNA, glycosaminoglycans (GAGs), and total collagen remaining within the decellularized larynx was undertaken for cartilage and muscle independently (mean ± SD; *p* value determined by 1‐way analysis of variance with multiple comparisons; *n* = 6 for each group). Statistical analysis was performed using Student's *t* test, for which *p* < .05 was considered statistically significant. The molecular data for each were as follows: cartilage: ∗, *p* = .0001; muscle: ∗, *p* = .0329 **(G)**; cartilage: ∗, *p* = .0001; muscle: ∗, *p* = .1099 (not significant) **(H)**; and cartilage: ∗, *p* = .002; muscle: ∗, *p* = .0001 **(I)**. **(G–I):** Both decellularized cartilage (*p* < .05) and muscle (*p* < .05) showed a statistically significant reduction in the amount of DNA. Similarly, the amount of GAGs remaining with the decellularized cartilage (*p* < .05) was reduced but preserved within decellularized muscle tissue. Collagen content was also significantly reduced in the decellularized cartilage and muscle tissue (*p* < .05 for each). Biomechanical analysis of the decellularized tissue components only revealed changes for the muscle but not cartilage. Abbreviations: CA, cartilage; CO, collagen; DC, decellularized; DC vac, decellularized using vacuum technology; H&E, hematoxylin and eosin stain; M, muscle; PSR‐ME, Picrosirius red with Miller's elastin; sGAG, sulfated glycosaminoglycan.

Our decellularized larynx was completely free of all cellular and nuclear material. Histological evaluation showed good preservation of tissue architecture and morphology of thyroid and cricoid cartilages, glandular tissue, muscle, and fine arterial elastin (Fig. 2A–2F). DNA content within muscle and cartilage was less than 50 ng/mg of tissue (Fig. 2G). Analysis of the ECM and its GAG content revealed a decrease, with 52% retention in the cartilage (cricoid and thyroid) and 46% in the muscle bundles (Fig. 2H). Quantitative collagen analysis showed a significant decrease of approximately 50% for cartilage and muscle. Histological evaluation under polarized light showed good structural integrity of collagen located within the cartilage and surrounding tissue (Fig. 2F). Biomechanical testing showed no significant difference between fresh and decellularized cartilage; however, a significant weakening of the muscle was noted (Fig. 2).

### In Vivo Assessment of the Seeded Graft

**Figure 3:**
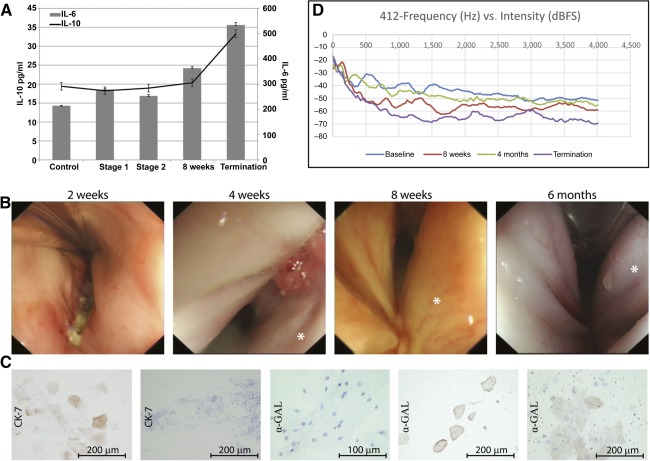
In vivo assessment of the implanted decellularized larynx over the duration of the study. **(A):** Blood serum levels of both IL‐6 and IL‐10. **(B):** Still bronchoscopy images at 2, 4, and 8 weeks and 6 months showing the mucosal surface. Each operated side is illustrated with an asterisk. Mucosal brushing and the subsequent staining of cells with Ck‐7 (left: normal human buccal cheek cells; second from left: mucosal brushing from animal 415) and α‐GAL (middle: human cheek cells; second from right: normal porcine cheek cells; right: mucosal brushing from animal 414). **(D):** A representative vocal recording profile (frequency vs. intensity) from animal 412. Abbreviation: IL, interleukin.

This study was designed to provide long‐term evidence of safety and efficacy of a seeded decellularized scaffold for partial laryngeal replacement. There were no clinical adverse effects due to the implanted scaffold; one pig was killed because of an ear infection relating to a long‐term underlying pathology exacerbated by immunosuppression. We saw no significant change in serum levels in either IL‐10 or IL‐6 over the duration of the study (Fig. 3B). The implanted scaffold provided a framework for tissue regeneration with good functional (i.e., airway; swallowing, which was not formally assessed, although feeding habit as part of the daily husbandry assessment was monitored together with weight gain) and vocal results.

We examined each animal over the course of the study to assess the macroscopic appearance of the mucosal surface on the operated side and the potential regeneration of the vocal folds. At the first bronchoscopy examination (2 weeks), a slightly inflamed but well‐vascularized mucosal surface was seen in each animal (Fig. 3B). In animal 417, there was closure due to swelling, but without tissue integration, at the anterior commissure. The macroscopic appearance of the implanted side did not differ from the contralateral (control) side in any animal. However, in all animals, we noticed the presence of a white material (possibly the implanted oral mucosal sheet), which had accumulated at the anterior commissure. This persisted, although it was reduced in amount at 4 weeks. By 6 months the white material was only present in animal 414. Vocal fold‐like features developed in each animal on the implanted side, although cranial to the normal cords on the opposite side. These folds were initially seen at 4 weeks as a depression and contouring of the mucosal surface, but by 6 months, they were well‐defined fold‐like features.

We could not identify human oral mucosal cells and the cell smears showed no positivity for human epithelial cells by CK7 staining, nor were any human cells of any phenotype identified with anti‐human MHC‐1 (data not shown) or anti‐human α‐GAL antibody. Porcine cells within the cell smears were positive for anti‐human α‐GAL (Fig. 3C) as early as 2 weeks after implantation.

For the vocalization analysis, qualitatively, we noticed a general hoarseness accompanied by an exaggerated roughness of varying degrees for each animal. Quantitatively, in four of the six animals, there was a shift in the peaks in the spectrogram of peak energies. For animals 413, 414, 415, and 416, the general shift in energy was from a lower frequency to higher frequency; for animal 412, this was reversed. In general, postoperatively, the areas of maximum spectral energy were between 500 Hz and 1,000 Hz, whereas the baseline recordings generally had maximum energy below 500 Hz and above 100 Hz (Fig. 3D).

### Ex Vivo Assessment of the Explanted Seeded Graft

**Figure 4:**
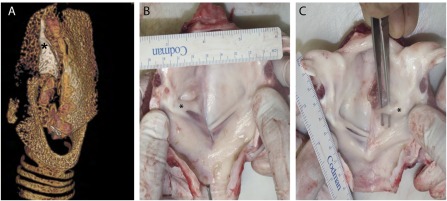
Ex vivo assessment of the implanted scaffold. **(A):** Static computed tomography image showing the remnants of the cartilage component (marked with an asterisk) of the decellularized scaffold; a three‐dimensional rotating image is presented in supplemental online Video 1. Representative images of the internal luminal surface showing good mucosal coverage and the development of a vocal fold (marked with an asterisk) **(B)** and a vocal “strap” of tissue **(C)**, both craniocaudally offset from the normal contralateral true vocal fold.

Due to limited contrast between the implanted seeded graft and native tissue, it was not possible to image via CT the implanted scaffold over the course of the study. However, following termination and explantation, each larynx underwent CT imaging to identify and locate the seeded graft. In each animal, the thyroid cartilage on the unoperated side was seen as a pitted sheet that was partially missing from the opposite side. Continued growth of this cartilage plate after surgery, without the counterbalancing cartilage on the opposite side, resulted in a slight rotation along the longitudinal axis toward the unoperated side, because the midline axis of the laryngeal prominence and the cricoid cartilage were no longer in alignment. In each case, distal to the laryngeal prominence, the tissue underwent rapid growth; this was especially evident in animals 415 and 416. In no animal was the intact implanted scaffold visible; however, in each case, white fragments of varying sizes (identified histologically as decellularized cartilage) of the scaffold could be seen; a static image is presented in Figure 4A, and supplemental online Video 1 presents a 3D rotating view.

Although the implanted cartilaginous component showed signs of fragmentation, the luminal mucosal surface showed significant regeneration. In each explanted larynx, on the implanted side, rudimentary vocal folds or straps had developed, extending from the arytenoid cartilage anteriorly. Animals 414 and 416 had well‐defined straps of tissue, whereas animals 412, 413, 415, and 417 showed regenerated vocal folds. In each case, as visualized on bronchoscopy, these were positioned higher than those on the opposite side. The size of the folds varied between animals (representative images are presented in Fig. 4B, 4C).

### Assessment of Epithelialization and Cartilage Remodeling

**Figure 5:**
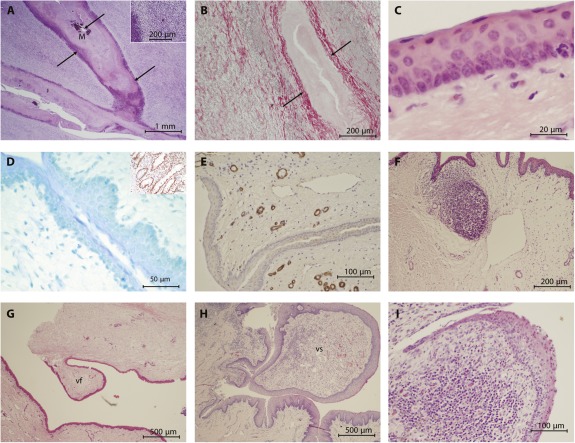
Epithelialization and mucosal coverage. **(A):** Remains of the tissue‐engineered oral mucosal sheet from animal 417 showing a concentration of inflammatory cells (insert) surrounding a mineralized center. **(B):** The collagen fibers of the original decellularized dermal collagen sheet (arrow heads) are more easily identifiable on the Picrosirius red with Miller's elastin‐stained section. In this image, the sheet has folded on itself and the paler central portion represents the original seeded aspect. Epithelialization of the mucosal surface showing stratified columnar epithelium **(C)** of porcine origin (insert shows positive stained human small intestine) **(D)**. **(E):** The underlying mucosa shows well established vasculature (α‐SMA stain). Both vocal folds **(G)** and strap **(H)** were identified histologically. **(I):** Tips of the vocal folds. Abbreviations: M, mineralized; vf, vocal fold; vs, vocal strap.

**Figure 6:**
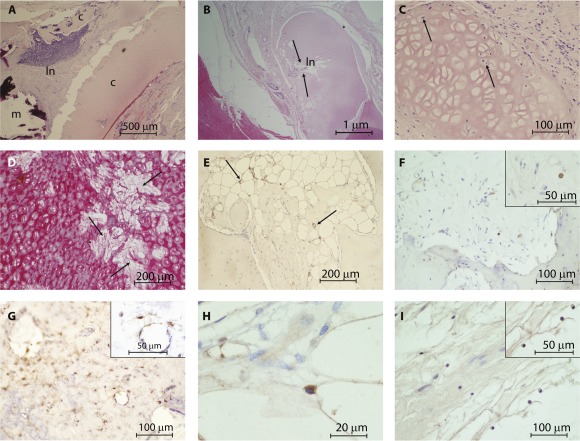
Cartilage remodeling. **(A):** Decellularized cartilage showing infiltration by a lymph node and mineralization, resulting in degradation. **(B, C):** This profile was not consistent across all animals; in some cases, the acellular cartilage was infiltrated with cells (arrows). **(D):** In addition, where cells had infiltrated the acellular cartilage, the cartilage was slowly remodeled into collagen fibrils (arrow). **(E):** Where cartilage was being remodeled, as in animal 412, the original decellularized cartilage had established blood vessels (stained with α‐SMA; arrows) and cells positive for collagen II (**F**; insert shows cytoplasmic positivity of a cell at higher magnification) CD 44 (**G**; insert shows a cell at higher magnification), and collagen X (**H**; cytoplasmic positive cell within the extracellular matrix). **(I):** Potential load‐bearing changes in the cartilage were illustrated by the presence of aggrecan cytoplasmic positive cells. Abbreviations: c, cartilage; ln, lymph node; m, mineralization.

Histologically, we could not identify the TE oral mucosal sheet in any explanted seeded graft. Animal 417 was killed early because of unrelated pathology; remnants of what might be the original seeded oral mucosal sheet could be identified, and appeared to elicit an immune response. The remains of the sheet were surrounded by a heavy inflammatory zone (Fig. 5A), the central portion of which showed collagen fibrils accompanied by considerable cellular debris (Fig. 6B), suggesting an adverse host response to the seeded sheet. By 6 months, in each animal, the mucosal surface on the operated side showed a contiguous epithelial layer consisting of stratified squamous epithelium (Fig. 5C), within which no cells were positive for Alu, confirming that all cells were host derived (Fig. 5D). Within the lamina propria, numerous blood vessels, both arterioles and venules of varying sizes (positive for α‐SMA), were located, as were well‐developed glandular tissue (both serous and mucinous) (Fig. 5E). Occasionally, there were either loose clusters of inflammatory cells (lymphoid aggregate) or well‐defined lymph node (i.e., mucosal associated lymphoid tissue) (Fig. 5F). In each animal, either a pseudo‐vocal fold (animals 412, 413, 415, and 417; Fig. 4G) or a strap of tissue (animals 414 and 416; Fig. 5H) had developed. Histologically, it was possible to distinguish between the regenerated vocal folds and the incompletely regenerated vocal straps. The tips of the folds showed signs of differentiation into more specialized cells (Fig. 5I).

In all animals, the thyroid cartilage component of the scaffold had displaced laterally from the original implanted site, probably due to the persistent pull of the sternohyoid muscle used to provide revascularization. However, in animals 413, 414, and 416, fragments of cartilage (thyroid and cricoid) from the original implanted decellularized scaffold could be identified, but they were remote from the initial surgically implanted site. In these cases, fragments of the acellular scaffold were infiltrated with different populations of cells surrounded by collagen fibrils, indicating remodeling of the hyaline cartilage together with evidence of angiogenesis.

In animal 413, there was a lymph node adjacent to the decellularized thyroid cartilage (Fig. 6A) next to which was an area of mineralization. However, this profile was not consistent across 3 of the animals (413, 414, and 416). The remaining cartilage in animals 416 and 414 was again predominantly acellular and there was little to no inflammatory response surrounding the cartilage (Fig. 6B, 6C). Instead, there was infiltration of fibroblast and macrophages into the implanted cartilage and small‐caliber blood vessels (stained with α‐SMA) (Fig. 6D, 6E). The ECM housing these cells consisted of collagen fibers rather than cartilage, suggesting remodeling of the original implanted tissue.

In animal 412, the original implanted acellular cartilage had relocated and was slowly being remodeled into bone; this was not the case in the remaining animals. In these animals the acellular cartilage showed signs of remodeling with collagen fibers. We probed the tissue for chondrocyte markers, both early; Collagen II (expressed by both early and mature chondrocytes) and CD 44 (cartilage homeostasis) and late collagen X (hypertrophic chondrocytes) (Fig. 6F–6H). In acellular cartilage not undergoing ossification, we found cells positive for aggrecan, indicating the initiation of chondrogenesis (Fig. 6I), in turn indicating the potential changes in the load‐bearing capability of the decellularized scaffolds. The fate of the seeded human BM‐MSCs could not be determined.

## Discussion

This study was designed to provide long‐term evidence of safety and efficacy (i.e., no long‐term physiological or functional impact) of a seeded decellularized scaffold for partial laryngeal replacement, with data suitable for submission for the award of a Clinical Trials Authorization (the U.K. equivalent of an Investigational New Drug in the U.S.). Through the nature of the target, unmet clinical need, and because our present grafts do not exhibit mobility, this study was not designed to replace a fully functional larynx but to assess whether a tissue‐engineered, full‐thickness partial laryngeal replacement could eventually be used to correct structural disorders. We have shown that it is possible to produce a biocompatible biological scaffold that has the potential to promote re‐epithelialization and submucosal regeneration. More importantly, it can provide the framework to maintain normal respiratory function, and appropriate swallowing and vocalization.

Previous tissue‐engineering strategies have used a scaffold‐based approach, with and without cell seeding, to restore structure and function. Decellularized scaffolds have been increasingly used in preclinical and clinical studies including the airway with some success 10, 11. Decellularization of the larynx has been reported using numerous methods, including enzyme based 8, enzymatic and detergent combinations 14, detergents combined with perfusion techniques 15, and freeze drying and sonication 16 for a number of different species. However, the use of negative pressure combined with hyper‐ and hypotonic solutions followed by enzymatic/detergents has not, to our knowledge, been previously reported. The introduction of negative pressure is likely to assist in drawing the reagents deeper into tissue more quickly and limit the damaging effects of the detergents and enzymes on the ECM. This approach took 8 days to produce a scaffold; a similar outcome was not possible in the same time frame if negative pressure was omitted. The enzyme‐based protocol used by Baiguera et al. 14 to decellularize a human larynx (similar in size to the pig) took considerably longer (25 cycles took longer than 2 weeks) and, therefore, our approach represents a potential financial savings and increases the possibility of producing a tailor‐made, off‐the‐shelf scaffold for laryngeal replacement.

In line with published studies, we have shown complete decellularization of the larynx with no disruption to tissue architecture and morphology, and with preservation of the collagen and elastin within the soft tissue and cartilage. However, the reduction in cartilage GAG content is likely to have contributed to the degradation and loss of mechanical durability of the thyroid and cricoid cartilage when implanted. The Blyscan Biocolor GAG assay does not give an indication of the structural integrity of the GAGs following decellularization (it simply gives the amount of sulfated GAGs) and, therefore, may not be appropriate as a proxy indicator of potential cartilage biomechanics in vivo. Additionally, we observed no alteration in tensile strength and rupture force for decellularized cartilage (similar to the reported human decellularized larynx 14), yet within 6 months, significant amounts of cartilage had degraded.

To date, implantation of fully tissue‐engineered larynx has not been undertaken, although human‐to‐human full laryngeal transplants have been successfully attempted 17. Implantations of vocal folds in a rat model using either a decellularized scaffold 8 or a multilaminar urinary bladder‐based ECM have been reported 18. Additionally, a porcine‐derived multilaminate ECM has been used for laryngeal reconstruction in dogs, resulting in cell‐ and tissue‐specific regeneration 19. In vivo studies in a large‐animal model using decellularized laryngeal scaffolds are limited. However, a submucosal placement of decellularized arytenoid into the body of the arytenoid in an equine model has been undertaken for 1 month 20. The authors reported no impact on respiratory function or swallowing during the course of the study, but the animal did remain intubated with a tracheostomy tube for 3 weeks of the 4‐week study (we did not intubate any animal postoperatively). Histological evaluation of the explanted tissue showed epithelialization accompanied by evidence of new hyaline cartilage formation but also complete degradation of the original decellularized scaffold.

In our study, we had a survival rate of greater than 80%: 5 of 6 pigs survived to planned death. The tissue‐engineered epithelial sheet could not be identified on bronchoscopic evaluation, although regeneration of the mucosal surface occurred in every animal. The lack of established vasculature may have contributed to the epithelium becoming necrotic and detached from the surface, and either coughed out or swallowed by the animal. However, the unplanned killing of 1 animal at 2 months postimplantation provided valuable information. Given that, the tissue‐engineered epithelial sheet in this pig could only be identified by the surrounding inflammatory reaction, yet an epithelial coverage had developed histologically. We hypothesize that it may not be necessary to use a tissue‐engineered epithelial sheet to promote re‐epithelialization. The previously reported equine study 20 also showed epithelial coverage without the use of a re‐epithelialization adjunct. However, comparison of adipose‐derived mesenchymal seeded and nonseeded tracheal scaffolds in rodents suggested accelerated re‐epithelialization 21, which is vital because airway tissue is prone to infection and mucous retention 22, 23.

Phonatory recordings showed changes in frequency and energy, but statistical correlation with morphological outcomes were difficult. Additionally, the lack of long‐term control data made interpretation of changes arising due to normal growth and development difficult; this is also the first time, to our knowledge, that pig vocalization has been objectively measured, so we lack normal reference ranges. Although we noticed an appreciable qualitative change in the tone of vocalization at the end of the study, it was impossible to determine how much of the vocalization was due to contribution from the unoperated side and how much was from the development of the new “pseudo” folds. Physical attributes relating to vocalization (e.g., “intensity, fundamental frequency, signal periodicity and harmonic to noise ratio” 24) rely on composition and mechanical stability of the tissue required to produce sound. It is possible that by keeping the decellularized vocal folds under constant tension (improved surgical attachment) and maintaining symmetrical height of the cords, we might have achieved a better anatomical outcome regarding both the positioning of the newly regenerated vocal folds and their anatomical appearance.

The cartilaginous component of the scaffold did not remain in its original anatomical craniocaudal location, due to either inadequate surgical retention or the additional tension caused by the sternothyroid muscle. In only one animal (pig 412) did we see evidence of ossification at the cut end of the native cartilage accompanied by vascularization within the decellularized cartilage. In this sample, within the decellularized component, we found cells positive for collagen type X, which is a characteristic feature of hypertrophic chondrocytes that play a pivotal role in endochondral ossification. During this process, a coordinated chain of events, starting with chondrocyte proliferation, maturation, and hypertrophy, takes place. In the latter stage, the hypertrophic cartilage undergoes calcification, there is an influx of vasculature, and the cartilage is slowly replaced by a bony matrix 25. However, endochondral ossification in human laryngeal cartilage has been documented 26; in contrast to endochondral ossification in growth plates, the hypertrophic chondrocytes and the resultant mineralized cartilage is not readily reabsorbed and can remain for many years 27.

In the acellular cartilage, we found few chondrocytes positive for both collagen II and aggrecan. The main proteoglycan secreted by chondrocytes is aggrecan and it provides the load‐bearing and compressive characteristics of cartilage. Collagen II is the major collagen secreted by chondrocytes and is specific for cartilage, where it provides the tensile strength required by this tissue. Cells positive for type II collagen are usually proliferating and differentiated chondrocytes 28, 29. Taken together, we hypothesize that the acellular cartilage, given time, would remodel as cartilage rather than bone, but the long‐term regenerative pathway is unknown. However, we did not probe for osteoblastic progenitor because we had not considered the possibility that the acellular cartilage would remodel as bone; we felt that the time frame would be too short. Additionally, the presence of cells positive for CD44 also suggests that acellular cartilage is being remodeled back into active tissue. CD44, together with integrin β 1, allows chondrocytes to function as mechanotransducers; as such, these receptors play a crucial role maintaining cartilage homeostasis 30. Our analysis of the acellular cartilage component of the implanted decellularized larynx is by no means comprehensive but merely an exploration of possible events likely to be taking place. Future work would need to concentrate on whether changes in the decellularized cartilage was initiated in response to the seeded mesenchymal stem cells or is purely a host‐derived progenitor cellular response.

## Conclusion

Patients who have had treatment for laryngeal cancer or the aftermath of trauma may be left with severe morbidity relating to swallowing, vocalization, and respiratory function; conventional therapies to address such structural disorders are suboptimal. This study was designed to provide long‐term evidence of safety and efficacy of a TE‐seeded decellularized scaffold for partial laryngeal replacement. We have shown that it is possible to decellularize an entire larynx and produce a biological scaffold that is safe and biocompatible, and has the potential to promote re‐epithelialization and submucosal formation. More importantly, our implanted scaffold provided the framework to maintain normal respiratory function together with appropriate swallowing and vocalization.

## Author Contributions

T.A.: conception and design, collection and/or assembly of data, data analysis and interpretation, manuscript writing; P.L., A.S., K.G., C.C., and L.P.: collection and/or assembly of data; A.B. and S.M.: provision of study material or patients; M.W.L. and P.D.S.: administrative support; M.A.B.: conception and design, financial support, and manuscript preparation.

## Disclosure of Potential Conflicts of Interest

M.A.B. has research funding from joint grants with Videregen. The other authors indicated no potential conflicts of interest.

## Supporting information

Supporting Information

Supporting Information
