# Supplementary material for: Stem Cell‐Based Tissue‐Engineered Laryngeal Replacement
Source: Stem Cells Transl Med. 2016 Sep 9;6(2):677–87. doi: 10.5966/sctm.2016-0130 (PMC5442815; doi:10.5966/sctm.2016-0130)
Supplement: Supplementary file 1 — Supporting Information [file SCT3-6-677-s001.pdf]

Supplementary material and video:

DNA quantification

In brief, 25 mg of minced wet tissue of fresh and de-cellularised laryngeal cartilage and muscle tissue (analysed separately) were placed in a micro-centrifuge tube with proteinase K and incubated in a water bath at 55°C for 4 hours with vortexing at 30 minutes intervals. Complete digestion was confirmed macroscopically and the samples were then subjected to a ribonuclease A solution at room temperature for 2 minutes. The samples were incubated with lysic reagents from the DNA extraction assay kit at 70°C for 10minutes. The lysates were loaded into prepared columns for binding DNA. After several washing steps to remove contaminates the DNA was finally eluted in 200µl of a Tris-ethyledeiaminetetraacetic acid solution. The absorbance was read at 260 nm and 280 nm using a self-masking quartz microcuvette and a spectrophotometer (Helios Alpha, Thermo Fisher Scientific, Loughborough, UK) and the absolute amount of DNA per milligram of tissue was calculated.

GAG quantification

In brief, 50 mg of minced wet tissue was placed in a micro-centrifuge tube and incubated with 1ml of papain digestion buffer at 65 ^{o}C for 18 hours. Aliquots of each sample were mixed with 1,9-dimethyl-methylene blue dye and reagents from the GAG assay kit. The absorbance at 656 nm was measured with a spectrophotometer and the absolute GAG content calculated by comparing to a plot of standards made from bovine tracheal chondroitin-4-sulfate.

Collagen quantification

In brief, 50 mg of minced wet tissue was placed in a micro-centrifuge tube with 1.5 ml acid-pepsin extraction medium (0.1 mg/ml pepsin in 0.5 mol/l acetic acid). Aliquots of each sample were incubated with acid-neutralizing reagent and collagen isolation reagents

overnight at 4°C. Samples were then subjected to the Sircol red dye from the collagen assay kit. The absorbance at 555 nm was measured with a spectrophotometer. By comparing to a plot of standards made from type I bovine skin collagen the absolute collagen content was calculated.

IHC analysis

Briefly, for IHC analysis 5μm paraffin sections were mounted on slides coated with (3-aminopropyl) triethoxysilane (Sigma-Aldrich, UK). Paraffin sections were de-waxed and rehydrated. Slides were placed in a humidification chamber and endogenous peroxidase was blocked using 3 % hydrogen peroxidase in methanol (Sigma-Aldrich, UK) for 30 minutes at room temperature. Any antigen retrieval required was performed. Non-specific binding sites were blocked with 2.5 % horse serum (Vector Laboratories Ltd., Peterborough, UK) at room temperature for 30 minutes. Sections were then incubated with the required antibody at the time and dilution specified above. After 3 x 3 minutes washes with PBS the sections were incubated with the secondary antibody (Impress anti-mouse or Impress anti-rabbit immunoglobulin IgG peroxidase kit, Vector Laboratories, Peterborough, UK) for 30 minutes at room temperature. After washing again (3 x 3 minutes PBS), the chromogenic substrate diaminobenzidine (Impact peroxidase substrate, Vector Laboratories, Peterborough, UK) was applied to the sections for 3 minutes at room temperature. After washing, the sections were counterstained with Harris's haematoxylin for 30 seconds before dehydrating, clearing, and applying a cover slip. For negative controls the same protocol was applied, however, the primary antibody was omitted and phosphate-buffered saline solution was used.

ALU detection

Briefly, sections were prepared as described above for IHC, deparaffinised in xylene (2x10minutes), hydrated in 100% ethanol and left to air dry. Following the manufactures

instructions a proteolytic treatment was applied for 15 minutes at 37°C followed by re-hydration through graded ethanol. Hybridisation procedure consisted of the application of a prepared probe (part of the kit), followed by denaturation for 10 minutes at 82°C. Slides were then kept in humidified chamber at 37°C overnight. The next morning the probe was washed off and a HRP- conjugate applied to the slides for 30 minutes at 37°C. Slides were then rinsed (3x1minute) in tris-buffered saline and incubated win AEC solution 5-15 minutes (until a colour change was observed). Slides were then rinsed with deionised water (3x1minute) and counterstained with methyl green and cover slipped using aqueous mounting medium.

Supplemental online video 1: 3D-Rotating Larynx- CT reconstruction showing the remnants of the cartilage component of the de-cellularised scaffold.
